# Developmental Assets, Self-Control and Internet Gaming Disorder in Adolescence: Testing a Moderated Mediation Model in a Longitudinal Study

**DOI:** 10.3389/fpubh.2022.808264

**Published:** 2022-02-04

**Authors:** Guo-Xing Xiang, Xiong Gan, Xin Jin, Yan-Hong Zhang, Cong-Shu Zhu

**Affiliations:** ^1^Department of Psychology, College of Education and Sports Sciences, Yangtze University, Jingzhou, China; ^2^Yangtze University College of Technology and Engineering, Jingzhou, China

**Keywords:** developmental assets, self-control, internet gaming disorder, longitudinal mediation model, adolescent

## Abstract

From the perspective of positive youth development, developmental assets and self-control play critical roles in promoting adolescent development. However, their effects have not been evaluated in the current issue, internet gaming disorder (IGD). IGD is gradually becoming an important social problem among worldwide youth and has been included in the eleventh International Classification of Diseases (ICD-11). Therefore, the present study was determined to investigate the relationship between developmental assets, self-control, and IGD. A two-wave longitudinal study, with each wave spanning half a year apart, was conducted in a sample of 1023 adolescents (aging from 11 to 15, 49.36% boys) during the COVID-19 pandemic. Results of the moderated mediation model revealed that T1 developmental assets could predict less IGD at T2 directly or through T1 self-control indirectly. Furthermore, the moderating effect of gender was not significant in the mediation model. Overall, adolescents who experience more developmental assets are less likely to suffer IGD. Moreover, developmental assets are conducive to developing a higher level of self-control, which in turn contributes to preventing or intervening in IGD as well. Therefore, measures should be taken to construct developmental assets to prevent or reduce IGD during adolescence.

## Introduction

During the COVID-19 pandemic, students are participating in online education through various mobile devices, such as computers and mobile phones (1). With these mobile devices, adolescents are more accessible to internet games, which may increase the possibility of internet gaming disorder (IGD). Moreover, the population of adolescents playing internet games is increasing around the world, meaning that more and more adolescents are facing the risk of IGD (2–4). IGD refers to uncontrollable, excessive, and compulsive use of internet games that leads to social and emotional problems (5). It is gradually becoming an important social problem among younger generations and has been included in the International Classification of Diseases (6). Meanwhile, a growing number of studies have demonstrated that IGD can lead to other developmental problems, including mental disorders and problematic behaviors (3, 7). More seriously, it will result in a series of alterations in brain function and structure (8–11). Considering the negative consequences of IGD, many researchers are devoted to figuring out the mechanisms, causes, and impacts of this phenomenon.

In addition to studying IGD's influence on individuals, researchers have made great achievements in explaining its reasons. Empirical studies have revealed that numerous factors significantly predict IGD, such as depression, anxiety, stress, aggression, peer attachment, social skills, family functioning, and parental monitoring (12–18). However, according to the ecological systems theory, Bronfenbrenner emphasized that different subsystems function as a whole to influence the growth of individuals (19). Thus, the combination of several factors can better explain the causes or mechanisms of IGD. Moreover, it is more in line with reality. Based on this theory, the present study aims to combine some factors to investigate their joint influence on IGD among adolescents. This will not only provide a further understanding of IGD but also contribute to the prevention and intervention of adolescents suffering from IGD among adolescents.

### Developmental Assets and IGD

In recent decades, developmental psychologists, inspired by positive psychology and comparative psychology, have paid increasing attention to positive youth development (PYD). This emerging perspective highlights that all young people have strengths and resources to effectively facilitate their healthy development (20). From this perspective, Benson et al. (21, 22) developed a universally used framework of developmental assets. Developmental assets are defined as 40 important resources that are conducive to positive youth development (23). According to the developmental assets framework (22), these 40 developmental assets can be divided into two categories: social resources (or external assets) and personal resources (internal assets). External assets comprise four kinds of resources, including support (e.g., family and social support, and positive family communication), empowerment (e.g., having responsibilities and chances to serve others and having a sense of safety), boundaries and expectations (e.g., having rules from family, school, and neighborhood and being expected to do well), and constructive use of time (e.g., spending time on creative and social activities). Similarly, internal assets also consist of four kinds of resources, including commitment to learning (e.g., looking forward to academic achievements and positive engagement in school), positive values (e.g., being honest, loyal, and responsible), social competence (e.g., having coping skills and social skills), and positive identity (e.g., self-esteem and having a purpose). These eight kinds of resources cover many assets from different subsystems, including individuals, families, schools, communities, and society. Previous research has confirmed that developmental assets can contribute to the positive growth of youth from various cultures (22, 24–26). Moreover, Chinese youth, with no exception, can also benefit from developmental assets under the perspective of PYD (27, 28).

The horizontal stacking assumption of the developmental assets framework stresses that experiencing as many kinds of resources as possible will prevent negative outcomes (22). Based on that, prior studies have indicated the protective function of developmental assets in risky behaviors among various countries' youth (29, 30). Encouraged by these findings, the present study aims to evaluate whether developmental assets could reduce or prevent IGD among Chinese youth. Empirical literature has provided evidence supporting the association between developmental assets and IGD indirectly. For instance, Bonnaire and Phan (31) found that family rules (included in boundaries and expectations) can effectively prevent boys from IGD. In a longitudinal study of Chinese adolescents, Su et al. (13) also revealed that parental monitoring (included in boundaries and expectations) plays a vital role in preventing IGD. In the same year, Kim et al. (12) reported that positive father-adolescent communication (included in support) is negatively associated with IGD among Korean adolescents. Similarly, Zhang et al. (32) indicated that social support (included in support) is negatively related to IGD symptoms among Chinese university students. Overall, all these findings from younger generations jointly indicate that IGD might be affected by social resources.

In addition, personal resources have been revealed to have negative impacts on youth IGD as well. Yu and his colleagues' longitudinal study (33) has revealed that the improvement of school engagement (included in commitment to learning) can significantly decrease IGD among adolescents. Moreover, Beard et al. (34) have found that self-esteem (included in positive identity) works as a protective factor to prevent IGD. Similarly, Zhang et al. (32) have indicated that purpose in life (included in positive identity) is an effective factor to protect university students against IGD. Recently, in a longitudinal study of freshmen, Teng et al. (16) have demonstrated that peer attachment (included in social competence) has a bidirectional association with IGD. In summary, both external and internal developmental assets are significantly associated with IGD in different countries' youth, including Chinese adolescents. Therefore, the present study hypothesizes that developmental assets are able to negatively predict adolescent IGD (Hypothesis 1).

### Self-Control as a Potential Mediator

Apart from the direct relationship between developmental assets and IGD, there may be another potential mediating mechanism. Self-control, a possible mediator, is universally considered as an ability to alter undesired behaviors or tendencies (35). High self-control capacity is always associated with positive developmental outcomes, such as successes in learning, relationships, and health (35, 36). However, people with low self-control abilities are more likely to face behavioral problems, including criminal problems and substance abuse (37–39). According to the general theory of crime, the most essential reason for crime and other problems is a lack of self-control ability (37). Özdemir et al. (40) and Li et al. (41) reported that low self-control can lead to internet addiction among undergraduate students and adolescents, respectively. Moreover, as a subtype of internet addiction, IGD has a similar association with self-control. Kim et al. (42) found that self-control can negatively predict IGD. Likewise, in a study of university students, poor self-control ability would lead to greater IGD (43). And among Korean adolescents, low self-control can significantly predict IGD (44). Considering the above theory and relevant findings, it is reasonable to believe that self-control ability might have a negative effect on adolescent IGD. That is, adolescents with a low self-control capacity may have more possibilities to suffer from IGD.

Gottfredson and Hirschi (37) have stated that self-control ability stays stable over a lifetime. However, more and more research puts forward evidence to the contrary that self-control changes during adolescence (45, 46). In addition to genetic effects, self-control ability will be shaped by other factors as well (47). Specifically, previous studies have demonstrated that self-control ability will be affected by developmental resources from different subsystems, such as teacher support, peer support, parental support, positive family communication, good parent-child relationship (included in support), parental supervision and monitoring, school discipline (included in boundaries and expectations) and self-esteem (included in positive identity) (48–53). Additionally, the ecological systems theory points out that subsystems will jointly impact individual development (19), which indicates that other developmental assets not mentioned above might also influence the self-control capacity. Therefore, it is reasonable to think that, as positive factors, developmental assets may also have a positive influence on adolescent self-control ability. Given the complicated association between self-control ability and developmental assets and IGD, the present study hypothesizes that self-control plays a mediating role in the association between developmental assets and IGD among adolescents (Hypothesis 2).

### Gender as a Potential Moderator

Previous evidence has shown that there are gender differences in developmental assets, self-control, and IGD. In a study of Indian youth, significant gender differences were found in both internal and external developmental assets (54). Similarly, Portuguese boys and girls experienced different developmental assets (55). A recent finding by Gomez-Baya et al. (56) reported that Spanish adolescents also face the same situation. According to the developmental assets framework (22), experiencing different developmental assets will have different influences on individual growth. Therefore, it is necessary to evaluate the possible gender differences in the present sample. Additionally, Jo and Bouffard (57) revealed that men and women experience similar developmental processes of self-control, but gender differences still exist over the short term. Likewise, Turner et al. (58) and Winfree et al. (59) both reported that women have higher self-control than men. Moreover, several studies have suggested that gender differences appear in IGD as well (60–62). Considering these differences between boys and girls, the present study hypothesizes that gender differences might also exist in the relationship between developmental assets, self-control, and adolescent IGD (Hypothesis 3).

### The Present Study

**Figure 1:**
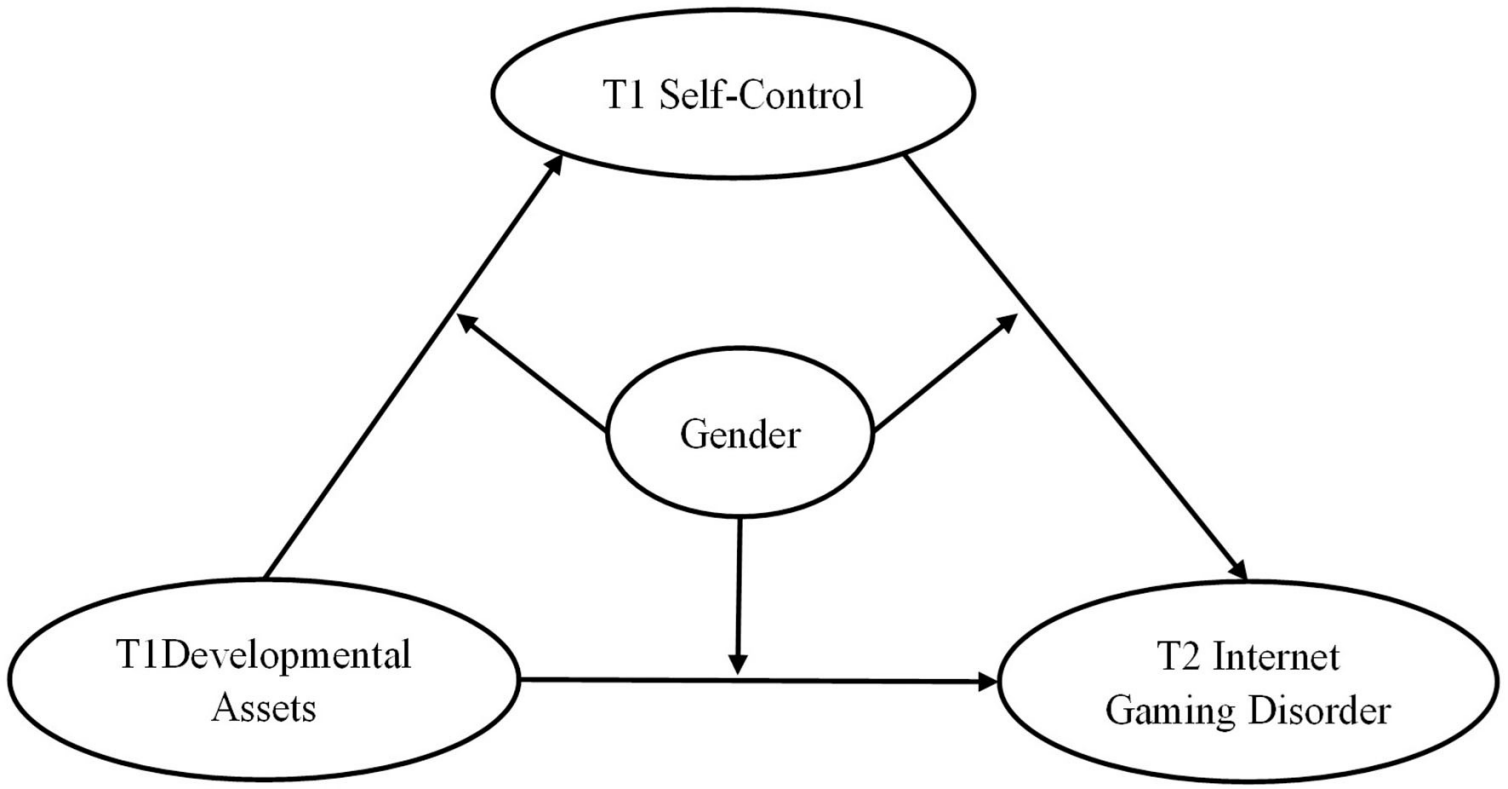
Conceptual moderated mediation model. T1 = Time 1; T2 = Time 2. The same below.

Although previous studies have made great achievements in the field of IGD, little research has considered this phenomenon from the perspective of PYD. With a combination of the developmental assets framework (22) and the general theory of crime (37) and existing evidence, we further realize the complex association between developmental assets, self-control, and IGD. Accordingly, the present study, based on the PYD, determines to investigate the relationship between developmental assets, self-control, and IGD among Chinese adolescents and constructs a moderated mediation model (see Figure 1). The present study hypothesizes that: (1) developmental assets can negatively predict adolescent IGD; (2) self-control will play a mediating role in the association between developmental assets and IGD among adolescents; and (3) gender differences might exist in the relationship between developmental assets, self-control, and adolescent IGD.

## Methods

### Participants and Procedure

Data was collected from two public middle schools in Southern China. By using a random cluster sampling, a total of 1,023 adolescents from Grade 7 to Grade 9 (505 boys; M_age_ = 13.16 years, SD = 0.86) were recruited to participate in the longitudinal study and complete the questionnaires. For various reasons, such as transfer, 30 participants dropped out (attrition rate = 2.93%). Most of the remaining adolescents were from families at the average economic level (3.10% were from families under the average economic level and 1.85% were from families above the average economic level). Moreover, the number of adolescents was about the same in three grades (35.75% were in Grade 7, 35.15% were in Grade 8, and 29.10% were in Grade 9). Additionally, Table 1 displays several descriptive characteristics of the current sample.

**Table 1:** Descriptive statistics of covariates and key variables.

	**Boys**		**Girls**		**Total**		
	***M***	***SD***	***M***	***SD***	***M***	***SD***	**Range**
**COVARIATES**							
1. Age	13.23	0.88	13.09	0.84	13.16	0.86	11–15
2. Family type	2.05	1.23	2.24	1.42	2.15	1.34	1–4
3. Family economic status	1.99	0.25	1.98	0.19	1.99	0.22	1–3
**KEY VARIABLES**							
4. T1 CDAI	2.60	2.42	2.486	2.35	2.54	2.38	0–8
5. T1 SC	40.13	7.03	39.82	6.96	39.97	6.99	17–63
6. T2 IGD	15.79	3.71	15.23	3.45	15.51	3.59	11–32

*CDAI, Cumulative developmental assets index; SC, Self-control; IGD, Internet gaming disorder*.

All procedures involving human participants were approved by the Research Ethics Committee of the College of Education and Sports Sciences, Yangtze University. After obtaining verbal consent from the school leaders and students, two researchers with negative test results for COVID-19 administered the questionnaire to participants, taking about half an hour during regular class time. Students were encouraged to self-report honestly by knowing about the anonymity of the questionnaires. The procedures were identical across waves, and the time interval of data collection was 6 months between two waves (Time 1, October 2020). The participation was entirely voluntary, and the students did not receive any compensation for their participation.

### Measures

#### Developmental Assets at Time 1

The present study used the Developmental Assets Profile (DAP) to measure adolescents' developmental assets (63). It consists of eight subscales, 58 items in total (e.g., “I have a school that cares about kids and encourages them” in the support subscale). All of these items were rated on a four-point scale, ranging from “1 = not at all or rarely” to “4 = extremely or almost always.” The composite of developmental assets came from the total scores of all subscales. This questionnaire demonstrated good reliability and validity among Chinese adolescents (64). In the present study, the Cronbach's alpha at time 1 was 0.95.

Inspired by previous literature (29, 64), the present study decided to construct the cumulative developmental assets index (CDAI) to reflect the breadth of developmental resources. Based on the average item response, this study chose “3 very or often” (indicating that students have many developmental assets) as the dividing point and binary recoded the eight subscales. More than or equal to the number of items in the subscale multiplied by three. The code of subscale scores was one to indicate that adolescents have this kind of developmental asset; otherwise, the code was zero to show that they do not have this kind of developmental asset. Then, add the scores recoded in each subscale to obtain the cumulative developmental assets index.

#### Self-Control at Time 1

The present study used the 13-item Brief Self-Control Scale (BSCS) (35) to assess adolescents' self-control (e.g., “Pleasure and fun sometimes keep me from getting work done”). And all of them were rated on a five-point scale (from “1 = not like me at all” to “5 = very much like me”). The composite score of BSCS came from the total scores of all items, with a higher score indicating better self-control ability. Among Chinese adolescents, this scale has good reliability and validity (65). Its Cronbach's alpha at time 1 was 0.71 in the present study.

#### Internet Gaming Disorder at Time 2

The present study used the 11-item Internet Gaming Disorder Questionnaire (IGDQ) (66) to measure adolescents' IGD (e.g., “Do you sometimes skip doing homework in order to spend more time playing online games?”). All items were rated on a 3-point scale, ranging from “1 = never” to “3 = frequently.” The composite of IGDQ came from the average scores of all items, with higher scores indicating higher levels of IGD. In Chinese adolescents, this questionnaire has good reliability and validity (66). Its Cronbach's alpha at time 2 was 0.77 in the present study.

#### Demographics

At both waves, the present study also collected several demographic variables, including adolescent age, gender (1 = boys, 2 = girls), grade, family type (1 = parents with one kid, 2 = parents with more than one kid, 3 = single-parent family with one kid, 4 = single-parent family with more than one kid), and family economic status (1 = under the average level, 2 = equal to the average level, 3 = above the average level).

### Data Analysis

Initially, the present study conducted the attrition analysis and common method bias analyses with SPSS 26.0. Second, descriptive statistics and correlations of the key variables were also calculated to assess the hypotheses 1. Third, structural equation modeling (SEM) and multigroup analysis were used to test the mediation model and the moderated mediation model in M*plus* 8.0 (67). To examine the hypotheses 2, a mediation model was constructed to assess the mediating effect of T1 self-control between T1 developmental assets and T2 IGD. Then, multigroup analysis was performed to test the hypotheses 3, the possible moderating effect of gender on the mediation model. Several model fit indices were evaluated in the present study, such as the values of Chi-square, degree of freedom, root mean square error of approximation (RMSEA; acceptable < 0.08, good < 0.05), comparative fit index (CFI; acceptable > 0.90, good > 0.95), Tucker-Lewis Index (TLI; acceptable > 0.90, good > 0.95) and the standardized root mean square residual (SRMR; acceptable < 0.08, good < 0.05) (68). Across the models in the present study, missing data was handled with full information maximum likelihood estimation (FIML) (69).

## Results

### Attrition Analysis and Common Method Biases Analyses

Prior to any further analysis, Chi-square tests and *t*-tests were conducted in SPSS 26, in order to examine the potential bias between participants who dropped out at time 2 and those who had provided data across two time points. The results showed that these two groups did not differ in T1 developmental assets [*t*_(28)_ = 0.96, *p* = 0.346], T1 self-control [*t*_(29)_ = 1.06, *p* = 0.299], T2 IGD [*t*_(1, 017)_ = −0.83, *p* = 0.409], gender [$\chi_{\left(1\right)}^{2}$ = 1.08, *p* = 0.298], family type [$\chi_{\left(5\right)}^{2}$ = 5.02, *p* = 0.413], and family economic [$\chi_{\left(2\right)}^{2}$ = 0.56, *p* = 0.756], revealing that the data set would not be biased due to attrition.

Second, to control the possible common method variance, the present study employed questionnaires with revering items and various types of scales response options (70). Besides, Harman's Single-Factor Test was also performed to test it (71). The results reported 25 factors with eigenvalues greater than one, and the first factor accounted for 16.19% of total variance, indicating that all results in the present study were less influenced by common method biases.

### Descriptive Statistics and Correlation Analyses

Table 2 outlines skewness, kurtosis, and intercorrelations of all variables. Specifically, T1 self-control was positively associated with T1 CDAI (*r* = 0.28, 95% CI = [0.219, 0.335]) and family economy (*r* = 0.10, 95% CI = [0.036, 0.164]), while it was negatively connected with T2 IGD (*r* = −0.19, 95% CI = [−0.256, −0.123]), and family type (*r* = −0.11, 95% CI = [−0.161, −0.043]). Additionally, T2 IGD was negatively associated with T1 CDAI (*r* = −0.11, 95% CI = [−0.172, −0.054]), family economy (*r* = −0.11, 95% CI = [−0.191, −0.017]), and gender (*r* = −0.08, 95% CI = [−0.136, −0.013]).

**Table 2:** Skewness, kurtosis, and intercorrelations of covariates and key variables.

	**Skewness**	**Kurtosis**	**1**	**2**	**3**	**4**	**5**
**COVARIATES**							
1. Gender	−0.01	−2.00					
2. Family type	1.10	0.51	0.07^*^				
3. Family economic status	−1.02	17.12	−0.01	−0.01			
**KEY VARIABLES**							
4. T1 CDAI	0.67	−0.67	−0.02	−0.05	0.05		
5. T1 SC	0.11	0.24	−0.02	−0.11^**^	0.10^**^	0.28^**^	
6. T2 IGD	1.71	3.68	−0.08^*^	0.04	−0.10^**^	−0.11^**^	−0.19^**^

*CDAI, Cumulative developmental assets index; SC, Self-control; IGD, Internet gaming disorder*.

* *p < 0. 05*,

** *p < 0.01*.

### The Mediation Effect of T1 Self-Control

**Figure 2:**
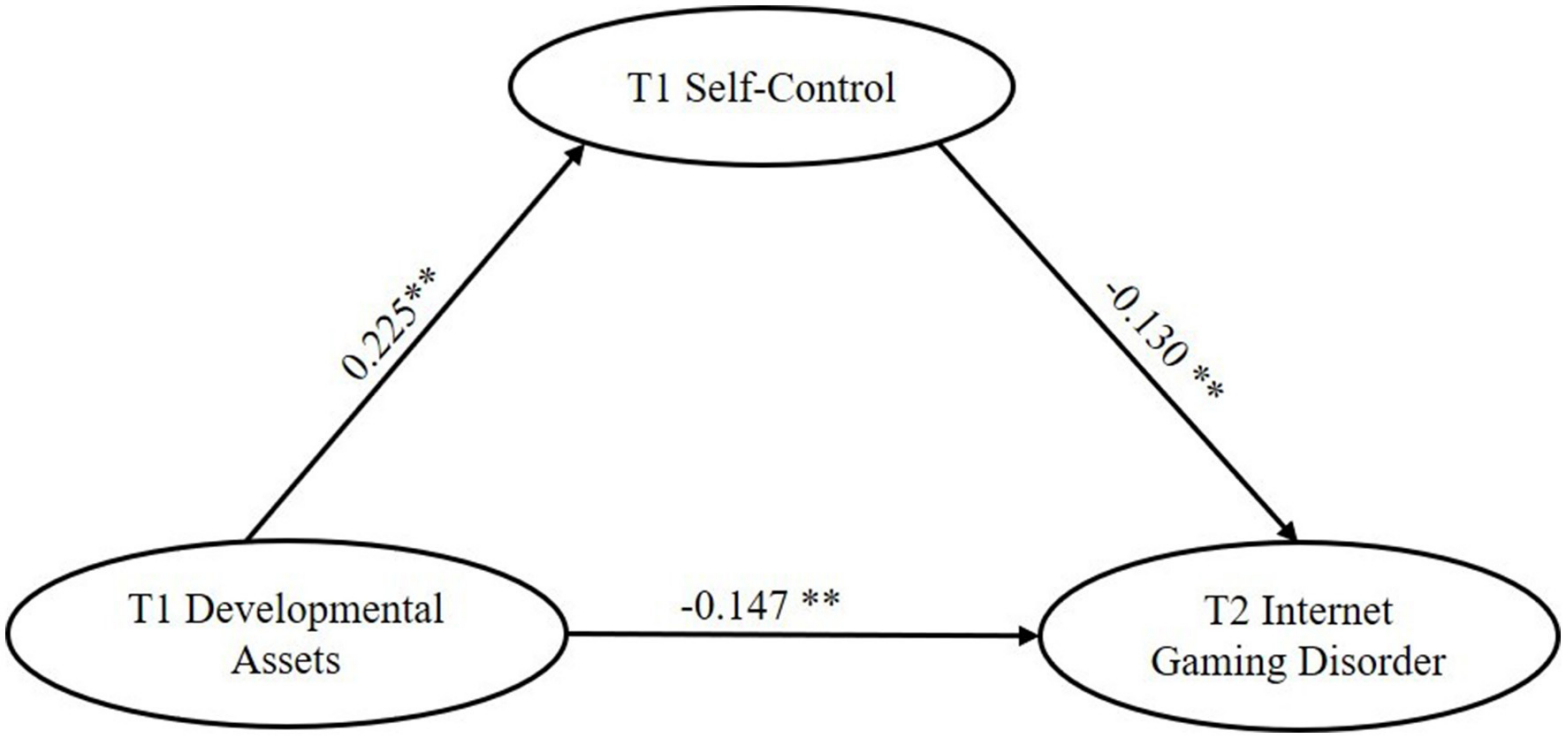
The mediating effect of T1 self-control in the relationship between T1 developmental assets and T2 internet gaming disorder. Standardized coefficients are reported; **p < 0.01.

A mediation model was constructed to test whether T1 self-control mediates the pathway from T1 CDAI to T2 IGD. And the results suggested that this model fit the data very well: $\chi_{\left(\text{df}\right)}^{2}$ = 70.37(33), RMSEA = 0.04 (95% CI = [0.026, 0.053]), CFI = 0.987, TLI = 0.981 and SRMR = 0.029. Furthermore, as illustrated in Figure 2, T1 CDAI could negatively predict the T2 IGD (β = −0.15, *p* = 0.005) and it could also predict T2 IGD through T1 self-control indirectly. Specifically, T1 CDAI positively influenced T1 self-control (β = 0.23, *p* = 0.001), which had a negative effect on T2 IGD (β = −0.13, *p* = 0.005). These results confirmed the mediation role of T1 self-control between T1 CDAI and T2 IGD (β = −0.03, 95% CI = [−0.066, −0.007]). That is, hypothesis 1 and hypothesis 2 were both confirmed.

### The Moderation Effect of Gender

Multigroup analysis was used to assess the possible moderation effect of gender on the mediation model. Based on the mediation model mentioned above, the present study added gender as a grouping item, and the new model also fit the data well: $\chi_{\left(\text{df}\right)}^{2}$ = 144.80 (60), RMSEA = 0.06 (95% CI = [0.045, 0.069]), CFI = 0. 967, TLI = 0. 960 and SRMR = 0.049. Then, Wald-Test was conducted to examine the possible gender differences in the mediation model. The results did not support the assumption of Wald-Test (χ^2^ = 1.011, *p* = 0.315), suggesting that there was no significant gender difference in the mediation model. Thus, hypothesis 3 was not supported.

## Discussion

To investigate the relationship between developmental assets, self-control, and IGD during adolescence, the present study adopts a two-wave longitudinal design and proposes a moderated mediation model. And the results generally confirm the hypothesized model. The major finding is that self-control mediates the association between developmental assets and IGD, which provides a new and comprehensive perspective for a deeper understanding of IGD among adolescents. Moreover, this finding indicates that various measures from different subsystems should be taken to prevent or intervene with adolescents suffering from IGD. The following paragraphs explain all the findings in detail.

### Developmental Assets and IGD

Previous studies have revealed that some specific contents of developmental resources are associated with IGD, such as social support, family rules, and school engagement (13, 31–33). Inspired by that indirect evidence, the present study aims to figure out whether they have a direct association. As expected, a significant relationship between developmental assets and IGD was disclosed directly through the longitudinal design. Specifically, developmental assets could negatively predict IGD half a year later. That is, experiencing as many developmental assets as possible could effectively reduce the risk of suffering IGD. Developmental assets play a protective role in IGD, which is in line with previous empirical evidence. For instance, Chang et al. (64) have found that developmental assets have cumulative effects on reducing externalizing behaviors among adolescents. Likewise, Mazloomi et al. (72) have also demonstrated that developmental assets could reduce addiction potential effectively. Overall, developmental assets serve as protective factors to prevent adolescents from having negative developmental outcomes.

Additionally, this finding also provides empirical support for the horizontal stacking assumption of the developmental assets framework (22). It stresses that experiencing as many kinds of resources as possible can prevent or reduce negative outcomes. In the present study, the CDAI was adopted to assess the cumulative effect of eight kinds of developmental assets on IGD. And results have suggested that the more developmental assets adolescents experience, the less likely they will be addicted to internet games. One possible explanation is the multiple protective functions (22, 64). Every kind of developmental resource is a protective layer that has its own unique effect and contributes to individual growth. The more developmental assets adolescents have, the more layers will protect their growth. The joint protective layers of developmental assets can form a giant umbrella, which in turn will stop adolescents from developmental problems and escort their healthy development. From the perspective of the developmental assets framework, constructing enough developmental resources is essential for the prevention and intervention of IGD and other negative outcomes.

### Mediating Role of Self-Control

Consistent with the second hypothesis, developmental assets could also decrease IGD indirectly through improving self-control. This finding provides longitudinal evidence for the ecological systems theory (19), which suggests that subsystems will jointly impact individual development. In the present study, developmental assets and self-control come from different subsystems such as individuals, families, schools, communities, and society. The significant influence of 40 resources and self-control on IGD supports the theory to a certain degree. Moreover, the present finding also corroborates the notion of the general theory of crime that lack of self-control ability is a critical reason for suffering IGD (37). Therefore, enhancing this ability could effectively decrease the prevalence of IGD among adolescents. Additionally, this finding is consistent with previous studies on the negative association between self-control and IGD (42, 44), but it also extends them by providing an actionable approach to improve the capability.

This finding can be more deeply understood with the combination of two theories. According to the developmental assets framework (22), developmental assets can promote positive developmental results (e.g., high self-control). Thus, experiencing as many developmental assets as possible is beneficial for the improvement of self-control. This is repeatedly confirmed by empirical studies (48–50). In addition, as highlighted in the general theory of crime (37), self-control is a vital capacity, the lack of which is the most essential reason for crime and other problems. In other words, improving self-control can effectively avoid or prevent crime and many problems, including IGD. Moreover, experiencing developmental assets is an effective and convenient way to increase the level of self-control ability. To sum up, developmental assets are helpful in developing a higher level of self-control, which in turn will prevent adolescents from suffering IGD. This finding deepens the understanding of the mechanism of the relationship between developmental assets and IGD. Therefore, various measures should be taken to provide as many developmental resources as possible in order to improve self-control ability.

### Moderating Role of Gender

Abundant empirical evidence has suggested gender differences in developmental assets, self-control, and IGD (55–57, 60–62). However, inconsistent with the literature, gender could not significantly moderate the mediation model in the present study. One possible explanation can be given for the insignificant moderating effect of gender. In the present study, CDAI was adopted as an indicator of developmental assets, which is a binary coded variable. And it may result in the loss of other important information about gender difference in the developmental assets. Soares et al.'s study (55) confirmed that gender differences were significant in the continuous distributions of developmental assets, while they were insignificant in the binary recoded index. Therefore, the binary recoding method might be an important reason. Future studies should cautiously adopt the binary recoding method and are encouraged to evaluate the gender difference with unrecorded scores of variables, which can reflect the situation more adequately.

### Limitations and Implications

It is necessary to consider several limitations of the present studies. First, the data was collected through self-reported questionnaires, which might include unreal answers and cannot avoid being influenced by shared method bias. So, multiple measures should be combined to investigate the relationship, for example, peer-reported questionnaires. Second, the present study only assessed the impacts of 40 developmental assets and one personality variable on IGD. However, developmental assets might include more factors. Future research could combine other factors to investigate their relationships with IGD. Third, although the present study adopted a longitudinal design, the time span is only half a year, which is too short in lifetime. Future research could adopt a longer longitudinal study. Last but not least, the present study only focused on Chinese adolescents. The findings might not be suitable for young people from other cultures. Therefore, in order to obtain more generalizable findings, future studies are encouraged to concentrate more on cross-cultural samples.

In terms of application, the present study has several implications. First, the present findings suggest that more attention should be paid to the positive factors (e.g., developmental assets) in adolescent growth. Second, comprehensive measures should be taken to construct development resources for adolescents to address the developmental challenges. For instance, parents can give more material and psychological support, communicate in a positive way, and build good relationships with adolescents. Their community and school can give them more opportunities to serve others and take on responsibilities. Third, self-control is an important ability that develops during the first decade and works through the lifetime to protect individuals. Therefore, parents have to consciously spare no effect to assist their children to develop this capability in the first decade of their lifetime.

## Conclusion

From the perspective of positive youth development, scholars indicate that developmental assets play a critical role in promoting adolescent development. The present study examined how their joint function affected the development of Chinese adolescents during the COVID-19 pandemic. Specifically, the present study explored the relationship between developmental assets, self-control, and IGD through a moderated mediation model based on a longitudinal study. Adolescents who have more developmental assets are less likely to develop IGD. Moreover, the cumulative developmental assets are beneficial to a higher level of self-control ability, which in turn contributes to preventing or decreasing IGD. In summary, measures should be taken to construct developmental assets to assist adolescents in responding to the developmental challenges during adolescence.

## Data Availability Statement

The raw data supporting the conclusions of this article will be made available by the authors, without undue reservation.

## Ethics Statement

The studies involving human participants were reviewed and approved by the Research Ethics Committee of College of Education and Sports Sciences, Yangtze University. Written informed consent to participate in this study was provided by the participants' legal guardian/next of kin.

## Author Contributions

XG designed the work. G-XX and Y-HZ collected the data. G-XX analyzed the data and drafted the manuscript. XG, G-XX, XJ, and C-SZ revised the manuscript. All authors contributed to the article and approved the submitted version.

## Funding

This research was supported by Youth Project of Natural Science Foundation of Hubei Province in 2020 (2020CFB365) and Achievements of key projects of education science plan of Hubei Province in 2019 (2019GA017).

## Conflict of Interest

The authors declare that the research was conducted in the absence of any commercial or financial relationships that could be construed as a potential conflict of interest.

## Publisher's Note

All claims expressed in this article are solely those of the authors and do not necessarily represent those of their affiliated organizations, or those of the publisher, the editors and the reviewers. Any product that may be evaluated in this article, or claim that may be made by its manufacturer, is not guaranteed or endorsed by the publisher.
